# Immune Thrombocytopenia Exacerbation After COVID-19 Vaccination in a Young Woman

**DOI:** 10.7759/cureus.17942

**Published:** 2021-09-13

**Authors:** Mai Fujita, Hiroshi Ureshino, Ayano Sugihara, Atsujiro Nishioka, Shinya Kimura

**Affiliations:** 1 Hematology Respiratory Medicine and Oncology, Saga University, Saga City, JPN; 2 Hematology, Oda Hospital, Kashima City, JPN

**Keywords:** young woman, high-dose dexamethasone, sars-cov2, pfizer biontech covid-19 vaccine, immune-mediated thrombocytopenia

## Abstract

The coronavirus disease 2019 (COVID-19) pandemic is one of the greatest health concerns worldwide. Safe and effective COVID-19 vaccines are urgently needed and have been rapidly approved. COVID-19 vaccine-induced thrombocytopenia was reported as a rare adverse effect in the Vaccine Adverse Events Reporting System. A 25-year-old woman, who was previously diagnosed with immune thrombocytopenia (ITP, stage I), had exacerbated severe thrombocytopenia (platelet count of 6,000/μL) with a headache, joint pain, general fatigue, and bleeding tendency three days after receiving her second dose of the Pfizer BioNTech COVID-19 vaccine. Pulsed high-dose dexamethasone therapy rapidly ameliorated the ITP. Although it is difficult to confirm a causal association between Pfizer BioNTech COVID-19 vaccination and ITP exacerbation, abrupt onset of ITP exacerbation after vaccination suggests that the ITP may be vaccination-induced thrombocytopenia exacerbation. Rare but severe adverse events such as ITP may be observed, depending on increased numbers of individuals who receive COVID-19 vaccines worldwide. Further investigation is needed to clarify the mechanisms of COVID-19 vaccine-induced ITP.

## Introduction

The numbers of coronavirus disease 2019 (COVID-19) cases are continuously increasing; more than 209 million cases and 4.4 million deaths were documented worldwide from the onset of the COVID-19 pandemic to August 2021 [1]; thus, the COVID-19 pandemic is one of the greatest health issues worldwide. Safe and effective COVID-19 vaccines are urgently needed, developed to fight the pandemic [2-4], and then they are approved by the Food and Drug Administration (FDA). COVID-19 vaccines were developed using new vaccine technologies that are messenger RNA (mRNA)-based or ﻿adenoviral vector (ADV)-based vaccines encoding the severe acute respiratory syndrome coronavirus 2 (SARS-CoV-2) spike protein. Although strict adverse effects are evaluated in clinical trials, rare but severe adverse effects are often documented after approval by the FDA’s Vaccine Adverse Events Reporting System.

Immune thrombocytopenic purpura (ITP) is a rare autoimmune disorder characterized by autoimmune platelet destruction with impaired platelet production. ITP has been documented as a result of vaccination through artificial immunization, and vaccination was reported as a risk factor for ITP exacerbation [5]. On the other hand, the incidence of COVID-19 vaccine-induced thrombocytopenia is estimated to be lower (0.80 per million doses) than the annual incidence of 3.3 ITP cases per 100,000 adults, which suggests that the incidence of thrombocytopenia cases after receiving COVID-19 vaccines is lower than the number of ITP cases expected [6]. We report a case of a patient with ITP who had exacerbated thrombocytopenia after receiving the Pfizer BioNTech COVID-19 vaccine.

## Case presentation

A 25-year-old woman who was previously diagnosed with ITP was admitted to our hospital because of severe thrombocytopenia (platelet count of 6,000/μL) and purpura in May 2021. She was often pointed out to have thrombocytopenia since childhood and approximately 1 year before (in May 2020), mild thrombocytopenia (platelet count of 78,000/μL) was documented by a medical checkup, and then thrombocytopenia (platelet count of 22,000/μL) developed in February 2021. She visited a prior hospital for a detailed examination of thrombocytopenia. At that time, a complete blood count showed mild thrombocytopenia, a white blood cell count of 7,900/μL (0% blasts), a hemoglobin level of 12.6 g/dL, and a platelet count of 85,000/μL. Laboratory tests revealed an elevated platelet-associated immunoglobulin G level (172 ng/10^7^ cells), while autoantibodies (antinuclear antibody, antineutrophil cytoplasmic antibodies, and rheumatoid factor) were not present. Anti-Helicobacter pylori antibodies were not present either. Bone marrow aspirate revealed the presence of megakaryocytes and preserved cellularity without leukemic blasts or dysplastic cells. G-banded metaphase analysis revealed a normal karyotype. The diagnosis of ITP was made, and the patient did not exhibit bleeding with a maintained platelet count (74,000/μL) in March 2021. She was under observation without treatment. Moreover, she previously experienced no seasonal flu vaccine-induced adverse events.

**Figure 1:**
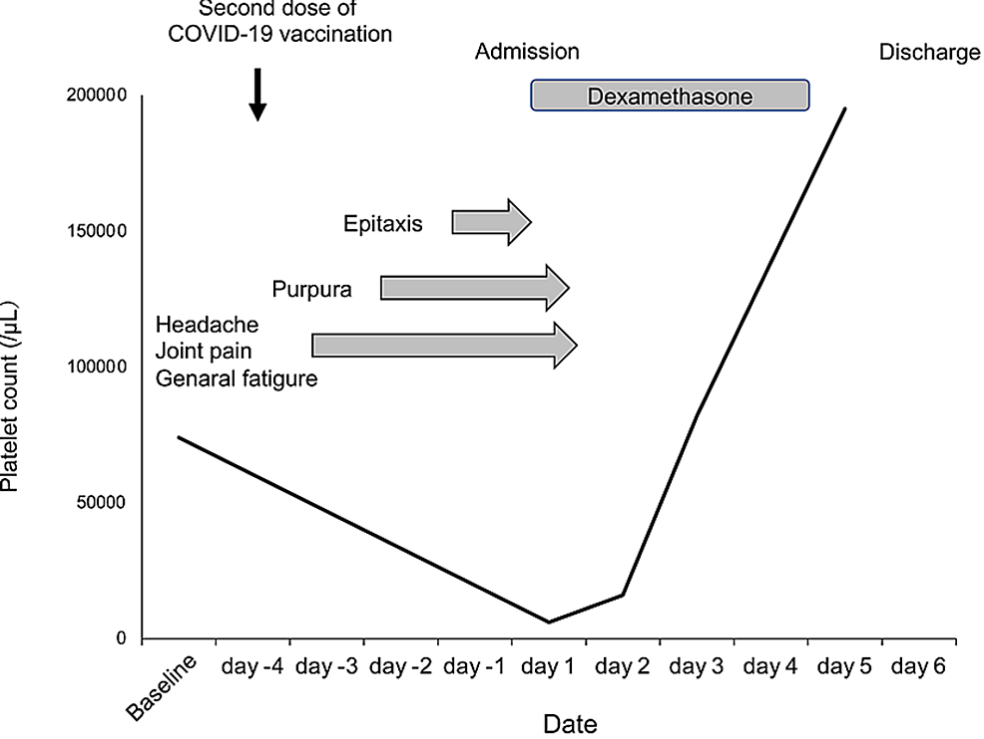
Clinical course of the patient

She received the first dose of the Pfizer BioNTech COVID-19 vaccine in April 2021. At that time, no vaccination-related adverse events were noticed. Three weeks later, she received the second dose of the Pfizer BioNTech COVID-19 vaccine in May 2021. The next day, she complained of a headache, joint pain, and general fatigue. Two days after vaccination, purpura appeared, and three days after vaccination, epistaxis occurred. She visited a prior hospital, and severe thrombocytopenia (platelet count of 2,000/μL) was documented. She was referred and admitted to our hospital in May 2021 (three days after vaccination). On physical examination, purpura was observed, while no hepatosplenomegaly or lymphadenopathies were observed. Laboratory tests revealed a slightly elevated white blood cell count (10,500/μL), normal hemoglobin level (12.4 g/dL) without schistocyte, and severe thrombocytopenia (platelet count of 6,000/μL) with an increased percentage of reticulated platelet (36.7%; reference range 2%-10%). The serum total bilirubin (0.4 mg/dL), aspartate aminotransferase (10 IU/L), alanine aminotransferase (6 IU/L), creatinine (0.5 mg/dL), and lactate dehydrogenase (147 IU/L) levels were all normal. Coagulation test revealed normal prothrombin time (12.7 sec), activated partial thromboplastin time (36.0 sec), fibrinogen (398.6 mg/dL), fibrin degradation product (2.7 mg/mL) and slightly elevated D-dimer (1.04 mg/mL) levels. Haptoglobin and ADAMTS13 autoantibody were not assessed. She did not receive recently started drugs (including heparin) and was not pregnant. These findings suggested the diagnosis of ITP exacerbation was made. Pulsed high-dose dexamethasone therapy (40 mg/day, oral intake, day1-4) was initiated, and the platelet count rapidly increased (82,000/μL on day 3; 195,000/μL on day 5), and then she was discharged on day 6 (Figure 1). She did not exhibit any respiratory symptoms and COVID-19 characteristic symptoms (abnormal olfactory and dysgeusia) in all clinical courses.

## Discussion

We report a patient with ITP who had exacerbated thrombocytopenia after receiving the Pfizer BioNTech COVID-19 vaccine. Although it is difficult to confirm a causal association between Pfizer BioNTech COVID-19 vaccination and ITP exacerbation, abrupt onset of ITP exacerbation and preceding systemic symptoms possibly associated with the immune reaction after vaccination, in this case, suggests that the ITP exacerbation may be drug (vaccine) induced.

﻿Drug-dependent autoantibodies that bind to platelets can generally cause drug-induced thrombocytopenia [7]. Likewise, artificial immunization following vaccination [against influenza virus, hepatitis B virus, human papillomavirus (HPV), and others] reportedly causes or exacerbates ITP. In the influenza vaccine, hemagglutinin is a viral surface glycoprotein that induces neutralizing antibodies following vaccination. Hemagglutinin can also bind to surface receptors of platelets, and antibodies against platelets can lead to autoimmune platelet destruction, possibly resulting in vaccination-induced ITP. Artificial immunization with hepatitis B surface antigen or the HPV L1 protein has resulted in cross-reactivity against a human peptide because ﻿the peptide is shared between hepatitis B surface antigen or HPV L1, and the human proteome. This cross-reaction possibly explains the pathogenesis of hepatitis B virus or HPV vaccine-induced thrombocytopenia [8].

There must be antigen(s) that can elicit an autoimmune reaction possibly associated with thrombocytopenia in this case, as well as for other vaccine-induced ITP. mRNA-based vaccines against SARS-CoV-2 are new vaccines that are different from conventional inactivated or live-attenuated vaccines. The vaccine consists of RNA encoding the SARS-CoV-2 spike protein encapsulated within a lipid nanoparticle (LNP). COVID-19 vaccine-induced allergic reactions have rarely been observed, possibly associated with LNPs, by the Vaccine Adverse Events Reporting System [9]. In the present patient, LNPs can elicit an immune reaction against platelets, resulting in vaccine-induced ITP exacerbation.

Unusual abnormal coagulation, including cerebral venous thrombosis and thrombocytopenia, which has resulted in death in some cases after receiving AstraZeneca (ChAdOx1 nCoV-19) or Johnson and Johnson (Ad26.COV2) vaccines, was reported. The rare but severe immune thrombotic thrombocytopenia was associated with antibodies specific to platelet factor 4 and was observed only in patients who received AstraZeneca (ChAdOx1 nCoV-19) or Johnson and Johnson (Ad26.COV2) vaccines, which are nonreplicating ADV-based DNA vaccines, not in those who received mRNA-based vaccines (Pfizer BioNTech or Moderna COVID-19 vaccines) [10,11]. Furthermore, anti-platelet factor 4 autoantibodies were detected even in patients who received mRNA-based vaccines [12], indicating that, although it is a rare event, vaccine-induced immune thrombotic ﻿thrombocytopenia can also occur, not only in patients who receive ADV-based COVID-19 vaccines but also in those who receive mRNA-based vaccines. In the present patient, obvious thrombosis was not identified, thus the pathology of thrombocytopenia is unlikely to be vaccine-induced thrombotic thrombocytosis.

The benefit of vaccination generally exceeds the risk of ITP exacerbation in patients with ITP. It may be reasonable to obtain platelet counts before and after vaccination. Then, if severe thrombocytopenia soon after vaccination (in the absence of other causes of thrombocytopenia) appears, it would be appropriate to initiate treatment (e.g. corticosteroid) for presumed vaccination-induced exacerbated ITP [13].

## Conclusions

mRNA-based or ﻿ADV-based vaccines against SARS-CoV-2 are new vaccine technologies. Rare but severe adverse events may be observed, depending on increased numbers of individuals who receive COVID-19 vaccines worldwide. Further investigation is needed to clarify the mechanisms of COVID-19 vaccine-induced immune thrombocytopenia.
